# The Relationship between Biofilm and Physical-Chemical Properties of Implant Abutment Materials for Successful Dental Implants

**DOI:** 10.3390/ma7053651

**Published:** 2014-05-07

**Authors:** Erica Dorigatti de Avila, Rafael Scaf de Molon, Carlos Eduardo Vergani, Francisco de Assis Mollo, Vehid Salih

**Affiliations:** 1 Department of Dental Materials and Prosthodontics, School of Dentistry at Araraquara, University Estadual Paulista-UNESP, 1680, Araraquara, São Paulo 14801-903, Brazil; E-Mails: vergani@foar.unesp.br; mollo@foar.unesp.br; 2 Department of Diagnostic and Surgery, School of Dentistry at Araraquara, University Estadual Paulista-UNESP, Araraquara, São Paulo 14801-903, Brazil; E-Mail: molon.foar@yahoo.com.br; 3 Peninsula School of Medicine & Dentistry, Plymouth University, C402, Portland Square, Drake Circus, Plymouth, Devon, PL4 8AA, UK; E-Mail: vehid.salih@plymouth.ac.uk

**Keywords:** biofilm, dental implants, titanium, zirconia

## Abstract

The aim of this review was to investigate the relationship between biofilm and peri-implant disease, with an emphasis on the types of implant abutment surfaces. Individuals with periodontal disease typically have a large amount of pathogenic microorganisms in the periodontal pocket. If the individuals lose their teeth, these microorganisms remain viable inside the mouth and can directly influence peri-implant microbiota. Metal implants offer a suitable solution, but similarly, these remaining bacteria can adhere on abutment implant surfaces, induce peri-implantitis causing potential destruction of the alveolar bone near to the implant threads and cause the subsequent loss of the implant. Studies have demonstrated differences in biofilm formation on dental materials and these variations can be associated with both physical and chemical characteristics of the surfaces. In the case of partially edentulous patients affected by periodontal disease, the ideal type of implant abutments utilized should be one that adheres the least or negligible amounts of periodontopathogenic bacteria. Therefore, it is of clinically relevance to know how the bacteria behave on different types of surfaces in order to develop new materials and/or new types of treatment surfaces, which will reduce or inhibit adhesion of pathogenic microorganisms, and, thus, restrict the use of the abutments with indication propensity for bacterial adhesion.

## 1. Introduction

The success of dental implants depends on the maintenance of osseointegration that is defined as a direct bone-to-implant contact without interposition of any other tissue [1]. Simultaneously, in order to preserve osseointegration around dental implants it is desirable to have no relationship between the maxillary and mandibular or parafunctional forces, mal-aligned forces of stress, peri-implantitis [2,3], absence of systemic diseases, e.g., diabetes mellitus [4], and to consider the host immune-inflammatory response to the bacterial challenge [5]. Despite the relatively high success rates of dental implant survival, reported to be higher than 90% for both partially or completely edentulous patients in longitudinal studies, some groups have demonstrated the role of putative periodontal pathogens in the etiology of peri-implantitis and their deleterious effects on hard and soft peri-implant tissues [6–10].

Late implant failure could be due to a disruption between implant and the mineralized tissues after osseointegration has been established due to overloading or microbial infection [11–13]. Whereas the main problem of osseointegration is solved by the use of high quality implants, with appropriate surface treatment and adequate surgical technique, the peri-implant tissue inflammation as a consequence of biofilms on abutments in the subgingival region is currently considered a major contributor to implant loss [14,15]. The presence of biofilms near to the implant abutments is characterized clinically by inflammation of the peri-implant mucosa progressing to subsequent destruction of the alveolar bone in contact with the implants threads. The teeth are unique structures, unlike the implants, which have the prosthetic restorations that bind to the implant body, e.g., crowns, metal structures or simple metal rods, which can lead to cracks or gaps forming between the implants and connectors. When compared to its natural non-implanted counterpart, peri-implant tissue comprises fewer fibroblasts, an increased amount of collagen fibers, blood supply, and the periosteal vascular plexus and parallel orientation of the gingival fibers [16].

In addition to these inherent factors in histopathology of peri-implant tissue, there are several differences in the designs of implants or macrostructure (screw *versus* cemented; one or two surgical stages), the type of surface or microstructure (commercially pure titanium, titanium alloys, titanium plasma sprayed, hydroxyapatite surfaces blasted with oxides, treated with acids, or a combination thereof) and the degree of smoothness or roughness or ultrastructure (crystallinity of the hydroxyapatite coating the implant, or nitrous acid type used), as well as different shapes and abutment materials [17]. Thus, these parameters existing between tooth and implant materials profoundly and directly influence the local microbiota.

## 2. Surface Characteristics of Abutments Implants

The scientific literature shows that bacterial plaque may play a prominent role as an etiologic factor responsible for implant loss after osseointegration, due to the presence of high levels of bacteria in the peri-implant sites [2,18–22]. As observed for teeth, the microorganisms need to interact with the implant abutment surface for the formation and growth of biofilm. Several studies suggest that some restorative materials have antibacterial activity, while others induce bacterial growth.

**Figure 1.**
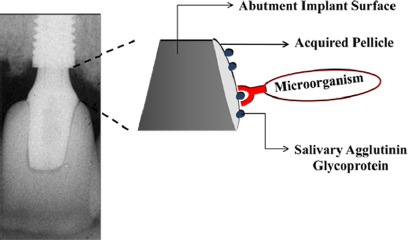
Image showing salivary acquired pellicle formation upon an implant surface as the first step in biofilm formation.

The physical and chemical characteristics of the materials will determine the type and quantity of the microbiota around these surfaces [23,24]. The non-specific physicochemical mechanisms of bacterial adhesion involve the superficial free energies and interaction surfaces theory in which adhesion is regarded as the interaction of Van der Waals forces and electrostatic phenomena [25]. Surface chemical composition, surface energy, surface water contact angle [26], and roughness are important parameters that may have a critical and fundamental influence on the interaction of biomaterial surfaces with proteins and cells. Once biomaterial surfaces have contact with biological molecules either *in vitro* or *in vivo*, the proteins present in the biological medium immediately coat the surfaces. Thereafter, salivary acquired pellicle formation takes place as the first step to biofilm formation (Figure 1).

With regard to the influence of surface roughness on biofilm formation, previous reports showed that protein adsorption and bacterial adhesion *in vivo* might be determined by a threshold surface roughness of 0.2 μm [27,28]. Burgers *et al.* [29] evaluated the initial biofilm formation, *in vitro* and *in vivo*, on different titanium surfaces and correlated these findings with different surface properties. Before biofilm formation, the authors determined the surface roughness and the surface free energy of samples and observed that the initial bacterial adhesion to differently textured titanium surfaces was primarily influenced by roughness surfaces values. This can be explained because the rough surfaces tend to entrap bacteria into micropits, protecting them from washing forces [28]. The difference in results from *in vivo*, *in situ*, and *in vitro* experiments is clear and the interfering factors involved are inclusion criterions established to select the patients, in relation to *in vivo* and *in situ* studies, and the number and types of bacteria used to biofilm formation, in case of *in vitro* study. Freitas *et al.* [30] in 2005 showed that a more rough surface causes an exponential increase in the number of bacterial cells, when just one kind of bacterium, *Streptococcus sanguis*, was utilized. However, when the study was performed upon the same type of surface, titanium, changing only the roughness value, and using a large number of bacteria species, the roughness does not act as an influential factor. In this case, no difference on bacteria adhesion can be justified by the same physical characteristic. The hydrophobicity and hydrophilic characteristic surfaces are other crucial elements that can directly influence bacterial adhesion [31]. In the case of implant surfaces, it is known that bone cells are attracted to a hydrophilic surface [32]. Recent studies have focused on the mechanism of chemical alterations within the dioxide titanium coating to enhance osteoconductivity and improve early osseointegration [33–35]. The increase in surface wettability may also have an influence on the amount of adsorbed proteins, since a very hydrophobic surface may prevent water from wetting the available surface, and, thus, further protein interaction with it. Alternatively, an increase in surface hydrophilicity may reduce the hydrophobic interaction between proteins and the surface, causing a lower adsorption affinity. Moreover, bacteria also have biomolecules in their cell wall that determine the surface properties and the adhesion dynamics [36]. In the case of gram-negative bacteria, the presence of lipopolysaccharide (LPS) in the outer membrane, tends to become more hydrophilic bacterial cell, and increase the attraction to hydrophilic surfaces too [37]. According to Husmark and Ronner, surface charge can also be influenced by the pH of the medium and consequently, change the bacteria adhered to it [38]. The relationship between surface and bacterial cell is mediated by a complex array of chemical and physical interactions, which add to the complexity of identifying the ideal surface with respect to abutment implants.

### 2.1. Types of Implant Abutments

In relation to the implant material types, titanium is the most commonly used material in dentistry due to its excellent physical and chemical characteristics, *i.e*., biocompatibility, stability and corrosion resistance [39]. To date, titanium is considered the “gold standard” and has maintained a dominant position as an abutment and implants material in long-term dental implant treatments. However, the high demand for aesthetic restorations has led to the introduction of ceramic implant abutments made from zirconium oxide stabilized with yttrium [40]. The microstructural and mechanical properties of the zirconia, as well as its excellent biocompatibility, have been well documented [41,42]. In dentistry, zirconia has been used for clinical applications in ceramic crowns, fixed partial dentures, orthodontic treatment supports, implants as well as abutments [43]. In addition, it has been shown that zirconia accumulates less plaque than titanium [42]. Despite the ceramic being used as abutment material for several years, only a limited number of related articles have been published concerning biofilm and abutment implants surfaces [44,45].

#### 2.1.1. Microbiology of Periodontal Disease

Periodontitis is a chronic inflammatory disease, initiated by the accumulation of plaque on enamel surfaces in close proximity with the gingival tissue, in which disease expression involves intricate interactions of the biofilm with the host immune inflammatory response and subsequent alterations in bone and connective tissue homeostases [46]. With the permanence of dental plaque on the tooth surface, the population dynamics of the microbiota is changed, favoring the development of biofilm with anaerobic bacteria, in particular microorganisms of the red complex (a group of bacteria that are grouped together based on their association with severe forms of periodontal disease) [47], which are responsible for alveolar bone loss and ultimately the tooth. Among periodontopathogenic bacteria, (*Porphyromonas gingivalis*), a gram-negative anaerobe and one of the most important pathogens in chronic periodontitis, has the ability for co-aggregation not only with (*Fusobacterium nucleatum*), but also with early colonizers (such as *Streptococcus gordonii*) [48], which could help explain its early appearance in the development of dental plaque biofilms [49,50]. However, it is important to mention that the virulence of *P. gingivalis* has been attributed to a variety of potential factors associated with its cell surface: fimbriae, lipopolysaccharides, capsules, proteases, hemagglutinins, and major outer membrane proteins [51]. On the tooth surfaces, these microorganisms are detected in dental plaque samples within six hours after professional tooth cleaning [52], and their numbers increase in compromised sites. Moreover, these structures can bind with receptors of epithelial cells, invade them and initiate an inflammatory process. The increase of cytokines released by the host defense cells can cause bone resorption and, consequently, loss of teeth or even implants. Attention has also been given to *F. nucleatum*, *a* gram-negative anaerobic bacteria, commonly found in the subgingival biofilm in periodontal pockets. This organism also has an important role in biofilm maturation, acting as a bridge between the early and late colonizers, guiding biofilm architecture and, consequently, enhancing the adherence of more periodontitis-associated bacteria [53]. As well as *P. gingivalis*, *F. nucleatum* is also capable of adherence to and invasion of host epithelial cells and stimulates the host immune inflammatory response. Since the presence of these microorganisms increases and/or decreases in the presence of other primary and intermediate colonizers, the successful treatment of periodontal disease would suggest an increase of the *Actinomyces spp*, and simultaneously, a reduction of pathogens of the orange and red complex [54].

#### 2.1.2. Periodontal Disease—Peri-Implant Disease

There is a philosophy that patients with periodontal disease should be considered a risk factor for peri-implantitis [55]. After partial alveolar bone loss as a consequence of periodontal disease, the periodontopathogenic microorganisms remain within periodontal pockets, and these microorganisms have the ability to colonize various implants even after osseointegration has been successfully achieved [47]. The remaining microorganisms adhere to the teeth, as well as on crowns and implants, and directly influence the peri-implant microbiota to promote the plaque development for a more subgingival microbiota [56–58]. The history of periodontitis has been associated with peri-implant disease. Marrone *et al.* [59] showed the prevalence of peri-implantitis in patients with active periodontitis was 57.1%. Thus, if a patient is not stable with respect to periodontitis they could have more chances to present peri-implantitis on one of their implants after >5 years duration. This finding is in agreement with a study regarding prevalence and risk variables for peri-implant disease in Brazilian subjects where those with periodontitis were more prone to develop peri-implantitis [60]. In addition, other studies have also associated a history of periodontitis with peri-implant disease [57,58,61]. Karoussis *et al.* [62] compared the survival rate of implants in patients with and without a history of periodontitis. They concluded that in 10 years, the implants survival rate for the group with a past history of chronic periodontitis was 90.5% while for the group with no past history of periodontitis was 96.5% [62]. Roos-Jansaker *et al.* [63] evaluated the long-term result of implant therapy, using implant loss as an outcome variable. The patients were called in for a complete clinical and radiographic examination, 9–14 years after implant placements. A significant relationship was observed between implant loss and periodontal bone loss of the remaining teeth at implant placement. Other authors associate the microbiota with unsuccessful healing of the implants [10,59,64–66]. What perhaps make such conclusions more difficult to interpret are the conflicting definitions of peri-implantitis found in the literature [60,67,68]. Depending on how peri-implantitis is defined, the frequencies of occurrence will considerably vary and it may become difficult for comparison between studies. Berglundh *et al.* [16] in a systematic review, reported frequencies of peri-implantitis ranging of 0% to 14.4%, with a weighted mean on fixed partial dentures of 6.4%. The authors observed that late implant loss (5–10 years) occurs in the range of 2.1% to 11.3%. This suggestion may be partly explain the controversial range of peri-implantitis and posterior implants loss. The bacterial colonization upon implant surfaces and in the gingival tissues may occur only minutes after implantation [69] and, after 10 days, the bacterial microbiota composition around these new implants becomes similar to microbiota around periodontally compromised teeth [70].

### 2.2. Biofilm Formation on Abutment Implant Surfaces

Strategies to reduce bacterial adhesion and biofilm formation on implant abutment surfaces play an important role on clinical practice and may be used to maintain soft tissue integrity or improve peri-implantitis treatment [71]. However, conflicting opinions exist on biofilm formation among different types of materials [15,72–75]. Titanium and zirconia are hydrophobic materials. Since gram-positive bacteria present hydrophobic characteristics due to a thick peptidoglycan layer, they will be attracted immediately to these materials. In contrast to gram-negative bacteria, those in direct contact will be repelled. Hydrophobic/hydrophilic interactions may explain why some reports do not show differences between biofilm formation when utilizing material surfaces of a similar chemical nature. Brakel *et al.* [76] compared the early bacterial colonization and the health of soft tissues adjacent to the mucosal surfaces of the titanium and zirconia abutments. Microbiological sampling and measurement of clinical parameters were performed two weeks and three months after abutment implantation. The authors concluded that there was no significant difference in bacterial adhesion in both abutments, titanium and zirconia [76]. Although titanium and zirconia are hydrophobic materials, titanium exhibits semi conductor features due to its bioactive dioxide layer [77] and this may explain controversial results in the scientific literature. Scarano *et al.* [15] showed that zirconia discs fixed on a device worn intraorally showed less plaque accumulation than Titanium discs, even with similar surface roughness. This was attributed to lower electrical conductivity of zirconia in comparison to titanium. Al-Ahmad *et al.* [75] also evaluated biofilm formation in various types of titanium and zirconia abutment surfaces *in vivo* and found that the oral biofilm accumulation was lower on the zirconia surface compared to the titanium surface.

It is also important to discuss, that when the implants are in contact with plasma or saliva, proteins can direct the attraction or repulsion of bacteria present on external layers since proteins have different degrees of hydrophobic to hydrophilic regions. The main salivary protein adsorbed to titanium *in vivo* and *in vitro* is albumin [78,79], and albumin adsorption to titanium occurs through calcium (Ca^+2^ bridges [80]. The negative charge from titanium dioxide may attract positive ions, such as Ca^+2^ and its presence thus increases the adhesion of some bacteria species. Hauslich *et al.* [81] 2012, demonstrated that pretreatment of titannium surfaces with Ca^+2^ ions increased the adhesion of *S. mutans* and *F. nucleatum* to the Ti surfaces, but did not influenced the *P. gingivalis* adhesion. *F. nucleatum* possesses Ca^+2^-dependent binding proteins on the cell surface similar to those of *S. mutans* [82]. These findings indicate that the divalent ion Ca^+2^ may serve as a bridging agent in the adhesion of bacteria to Ti surfaces.

Bacteria can detect the non-biological substrate and express different genes, probably as part of the adaptation to a new microenvironment. The differences in the depth and viability of the biofilms on the different materials are a result of physical and chemical properties that determine gene expression profiling of bacteria, regardless of film formation [75].

## 3. Conclusions

In the case of partially edentulous patients affected by periodontal disease, the type of abutment implant to be used requires careful consideration. In general, previous reports compare biofilm formation on different types of surfaces using few numbers of bacteria. The multiple factors involved in complex biofilm formation, such as roughness and electrostatic interactions between bacteria and surfaces and interbacterial interactions can make it difficult to characterize and determine the ideal abutment implant surface. However, understanding the influence of materials surfaces on bacterial adhesion will help future development of new materials or surface treatments, in order to reduce or inhibit adhesion of pathogenic microorganisms on them.
